# Successful localisation of recurrent thyroid cancer using preoperative patent blue dye injection

**DOI:** 10.1007/s40618-024-02301-w

**Published:** 2024-02-14

**Authors:** B. O. Evranos, N. Ince, H. Ataş, S. B. Polat, H. Ahsen, N. N. Imga, A. Dirikoc, O. Topaloglu, T. Tutuncu, R. Ersoy, B. Cakir

**Affiliations:** 1 Faculty of Medicine, Department of Endocrinology and Metabolism, Ankara Yildirim Beyazit University, Ankara Bilkent City Hospital, Ankara, Turkey; 2 Faculty of Medicine, Department of Surgery, University of Health Sciences, Ankara Bilkent City Hospital, Ankara, Turkey; 3 Department of Pathology, Ankara Bilkent City Hospital, Ankara, Turkey; 4 Faculty of Medicine, Department of Endocrinology and Metabolism, University of Health Sciences, Ankara Bilkent City Hospital, Ankara, Turkey

**Keywords:** Patent blue, Blue dye, Thyroid cancer recurrences, Localisation, Reoperative surgery

## Abstract

**Purpose:**

In the follow-up of patients with thyroid cancer, recurrences are often detected, posing challenges in locating and removing these lesions in a reoperative setting. This study aimed to assess the effectiveness of preoperative ultrasound (US)-guided injection of patent blue (PB) dye into the recurrences to aid in their safe and efficient removal.

**Methods:**

In this retrospective analysis, we reviewed the records of the patients in a tertiary care centre between February 2019 and March 2023 who underwent US-guided PB injection in the endocrinology outpatient clinic before reoperative neck surgery. The duration between the injection of PB and the initiation of surgery was recorded. The complications and effectiveness of the procedure were evaluated using ultrasonographic, laboratory, surgical, and pathologic records.

**Results:**

We reached 23 consecutive patients with 28 lesions. The recurrences averaged 8.8 mm (4.1–15.6) in size and were successfully stained in all cases. The median time between the PB injection and the incision was 90 (35–210) min. There were no complications related to the dye injection. The blue recurrences were conveniently identified and removed in all cases.

**Conclusions:**

A preoperative US-guided injection of PB is a safe, readily available and highly effective technique for localising recurrent tumours, even in small lesions within scarred reoperative neck surgeries.

## Introduction

Thyroid cancer stands as the predominant endocrine malignancy, with a notable surge in its incidence over recent decades [1]. Differentiated thyroid cancer (DTC), which includes papillary thyroid carcinoma (PTC) and follicular thyroid carcinoma (FTC), stands out as the most prevalent among various types of thyroid cancer. It accounts for over 90% of all cases [2, 3]. Poorly differentiated thyroid carcinoma and anaplastic thyroid carcinoma (ATC) are infrequent tumours, accounting for 5% and 1%, respectively. These cancers exhibit aggressive behaviour and have a relatively short median survival time of 5 years and 6 months, respectively. In contrast, medullary thyroid carcinoma (MTC), constituting 5% of thyroid cancer cases, originates from parafollicular C cells.

Despite the generally favourable prognosis of DTC, 6–30% of patients experience persistent disease or recurrence [4, 5]. Neck lymph nodes emerge as the predominant site of recurrence [6]. MTC is relatively uncommon; local recurrence or persistent disease poses a common challenge, affecting up to 79% of patients during extended follow-up periods [7].

Surgery represents an offered treatment choice for cervical persistent or recurrent DTC under suitable conditions [8]. A strategically planned neck dissection should be contemplated, addressing the involved nodal compartment, central (levels VI and VII) and/or lateral (levels II–V) while preserving uninvolved critical anatomical structures. In instances where the patient has previously undergone a comprehensive neck dissection, the objective is to ensure the adequate excision of the lesion [8, 9].

The decision to recommend surgery for recurrent nodal disease in the neck involves a careful balance between two conflicting considerations: the heightened risks associated with revision surgery, usually surpassing those of primary surgery due to scarring from prior procedures, and the acknowledgement that surgical resection is typically the most effective treatment for macroscopic gross nodal disease compared to alternative treatment options [10]. The availability of expertise in revision thyroid cancer nodal surgery is a crucial factor in this decision-making process. Techniques that aid in the surgeon's localisation of the index lesion enhance the convenience of surgery [11]. The guidelines endorsed by the American Thyroid Association (ATA) recommend surgical intervention when central neck compartment nodes exceed 8 mm or lateral neck nodes surpass 10 mm [8]. Smaller lesions are probably best managed with active surveillance (observation), serial imaging, serum tg measurement reserving FNA, and subsequent intervention for documented structural disease progression. However, in addition to size, multiple factors should be assessed when considering surgical options, including the proximity of given malignant nodes to adjacent vital structures and the functional status of the vocal cords. Patient comorbidities, motivation, and emotional concerns should also be taken into account along with primary tumour factors (high-grade histology, rapid Tg doubling time, RAI avidity, 18FDG-PET avidity, and presence of molecular markers associated with aggressive behaviour) [8]. Adjuvant RAI therapy is recommended for high-risk and selected intermediate-risk patients [8]. Surgical and pathological reports guide RAI decisions. RAI-refractory lesions pose challenges, necessitating multidisciplinary decisions. External beam radiation therapy (EBRT) is considered for highly selected cases, serving as adjuvant therapy or definitive treatment for unresectable relapses [12]. EBRT is an option when surgery or RAI is not feasible, considering the patient's context. Its role remains controversial, and careful risk–benefit analysis is crucial. Ultrasound (US) guided percutaneous ablation is a localised treatment for DTC patients with localised lymph node metastases, suitable for poor surgical candidates [13]. Thermal and chemoablation techniques show reasonable results, but risks include local complications. Tyrosine kinase inhibitors like lenvatinib and sorafenib are crucial in managing unresectable and RAI-refractory lesions [14]. They improve progression-free survival but are not curative. Careful patient selection and ongoing research into resistance mechanisms contribute to improved outcomes. For cases of MTC with locoregional disease and no distant metastasis, the primary treatment option is surgical resection [15].

Recurrences are frequently found within regions that have been previously operated. Previous surgeries can lead to significant scarring and deformities in the anatomy of the neck. This can create challenges in accurately identifying the recurrent laryngeal nerve and parathyroid glands, which raises safety concerns. Due to these difficulties, performing neck reoperations can be technically compelling and may lead to increased patient risks and complications [16]. Considering the small dimensions of numerous recurrences, differentiating between tumour and scar tissue can frequently pose a significant hardship. Consequently, some patients may undergo unsuccessful surgical attempts at removing the tumour. Furthermore, it is essential to note that even with the removal of lymph nodes, there is still a chance that the index node identified before the surgery may be missed.

Hence, there is a need for precise and convenient preoperative localisation to enhance surgical outcomes. Several methods have been employed to achieve preoperative localisation of the identified lesion, including US lymphatic mapping [17], intraoperative US [18, 19], wire-guided localisation (WGL) [20], intraoperative US-guided dye injection [21], radio-guided occult lesion localisation (ROLL) [22–24], radioactive seed localisation (RSL) [25, 26], and US-guided charcoal injection [27, 28]. Each of these methods possesses different efficiencies and challenges.

This study aimed to assess the safety and efficacy of preoperative US-guided injection of patent blue (PB) dye for localising recurrences following thyroidectomy.

## Materials and methods

A retrospective review was conducted on the medical records of patients at Ankara Bilkent City Hospital who underwent preoperative injection of PB dye to localise nonpalpable recurrences before reoperative neck surgery. The study was conducted after obtaining approval from the hospital's ethics committee.

The metastatic presence in any cervical lymph node or residual thyroid tissue is defined as a recurrence. In all cases, a diagnosis of thyroid cancer recurrence was confirmed through US-guided fine-needle aspiration (FNA). All study participants satisfied the criteria for US-dye injection, including nonpalpable recurrence in the neck region, a history of prior thyroid cancer surgery, detectable recurrence in the US, and accessibility via percutaneous approach. Between February 2019 and March 2023, we identified 23 consecutive patients with 28 lesions. All lesions were assessed by the US after surgery.

**Fig. 1:**
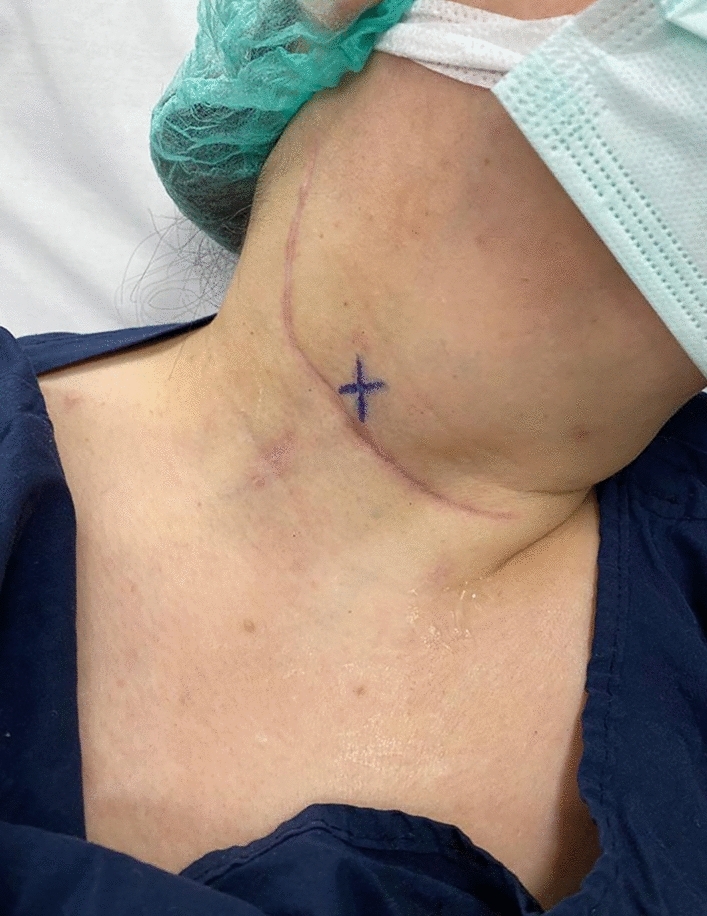
Injection site was marked with a pen

The patients were taken to the endocrinology outpatient clinic for US-guided PB administration before surgery**.** All PB dye injections were performed by the same two experienced endocrinologists at our institution who had also completed the patients’ FNA. The patient was supine with their neck hyperextended to provide optimal visibility of the recurrence. After the recurrent lesion was identified using the US, careful consideration was given to planning the safest and most efficient route to prevent any accidental puncture of major blood vessels in the neck. The area was cleaned with an alcohol solution. A tuberculin syringe (1 ml) and 23–25-gauge needles were used for injection. The needle tip’s position within the lesion was verified with real-time US guidance, and 0.3–0.5 ml of PB solution was injected. Only one injection was given directly into the lesion**.** After withdrawing the needle, the injection site was marked with a pen (Fig. 1). Subsequently, the patients were transferred to the operating room, where the planned surgical procedures were carried out following the administration of anaesthesia. The timing of the PB injection and the initiation of the incision by the surgeon were extracted from the records, allowing for the calculation of the duration between dye injection and incision.

The initial surgery was meticulously documented, capturing essential details such as pathology findings and the spectrum of operations performed, which included total thyroidectomy (TT), central lymph node dissection (CLND), and lateral lymph node dissection (LLND). A thorough analysis of pathology reports was conducted to determine TNM staging following the AJCC/UICC 8th edition guidelines [29]. In addition, patients were stratified into a three-tiered clinicopathologic risk classification system, categorising them as low, intermediate, or high risk of recurrence based on the 2015 ATA thyroid cancer guideline [8]. Hobnail or tall cell variants were classified as aggressive variants.

A detailed assessment was conducted to quantify the number of lymph nodes extracted, called lymph node yield (LNY), in both CLND and LLND specimens. Identifying and documenting malignant lymph nodes were crucial for subsequent computation of the lymph node ratio (LNR), determined by the ratio of malign lymph nodes to LNY. US findings and the number of prior thyroid surgeries before the staining procedure were also recorded.

In cases involving papillary thyroid carcinomas (PTCs), comprehensive documentation included thyroid-stimulating hormone (TSH), thyroglobulin (Tg), and anti-thyroglobulin antibody (anti-Tg) values in both the preoperative and postoperative phases. For cases with medullary thyroid carcinoma (MTC), diligent recording extended to encompass calcitonin (CT) values. Serum levels of Tg, anti-Tg, CT, and TSH were assessed using electrochemiluminescence immunoassay methods. The established normal ranges for these measurements were as follows: 1.6–60 μg/L for Tg, less than 1.3 IU/mL for anti-Tg, less than 5 ng/L for CT, and 0.55–4.78 mU/L for TSH.

Information was collected regarding whether patients underwent radioactive iodine (RAI) treatment before and during the follow-up after the staining procedure. Post-surgery, a comprehensive record was maintained, encompassing the spectrum of surgery (CLND, LLND, selective lymph node dissection (SLND), and excision (E)), the pathology and the results obtained from the postoperative US. SLND (levels IIa, III, IV, Vb, and VI) or E (index lymph node) was performed if the targeted neck level had been previously dissected. Successful resection of the recurrence was determined by postoperative pathology, confirming the presence of malignancy in the stained lesion, along with postoperative US ensuring the elimination of the index lesion. The study documented the duration of follow-up, identification of the index lymph node through the US, and the measurement of Tg and anti-Tg or CT levels during the follow-up period.

The locations of recurrences and the maximum diameter of the stained lesion observed in the US were recorded. The site of a cervical recurrence was described according to the general arrangement of the lymphatic systems in the head and neck area [30]. Regarding the safety of PB injection, whether there were complications such as anaphylaxis, intolerable pain, bleeding, nerve damage, blue skin staining, and skin or fat necrosis at the injection site was recorded.

## Results

The study included eight men and 15 women aged 21–76, with a median age of 41. Table 1 presents a detailed overview of the baseline characteristics for all cases separately, whereas Table 2 provides patients’ general features. The initial surgeries revealed PTC in 21 and MTC in two patients. Among the initial surgeries, TT was performed in 8 (34.8%) patients, TT with CLND in 4 (17.4%) patients, and TT with CLND and LLND in 11 (47.8%) patients. The detailed TNM staging is presented in Table 1. Notably, none of the patients exhibited metastases. Twenty patients, constituting 87% of the cohort, were classified as TNM stage I, while three patients, comprising 13%, were categorised as TNM stage II. The evaluation of ATA risk was limited to 20 patients with PTC, given that the initial operation for one patient occurred in another centre 20 years ago, and the specific details of the pathology were unavailable for assessment (Tables 1 and 2). One patient, constituting 5% of the cohort, fell within the low-risk category, while nine patients (45%) were classified as intermediate risk, and ten patients (50%) were designated as high risk. The median number of tumour foci was 2, ranging from 1 to 9. Among 20 patients with PTC, nine (45%) individuals exclusively presented with classic variants, two (10%) with solely follicular variants, and three (15%) with only aggressive variants. Notably, multifocality revealed classical and follicular variants coexisting in four (20%) patients, classical, follicular, and solid variants in one (5%) patient, and both classical and aggressive variants in another one (5%). Table 1 also displays information regarding both malign lymph nodes and LNY. LNR median (range) was 0.23 (0–0.71).

**Table 1:** Baseline characteristics of 23 patients who underwent reoperation after localisation using ultrasound-guided patent blue injection

	Age	Sex	First operation	Pathology	Foci	Variant	Malign LN/LNY	LNR	TNM	TNM Staging	ATA risk
Case 1	54	M	TT + CLND + LLND	PTC	4	Classic/ Follicular/Solid	21/52	0.40	T3bN1b	I	High
Case 2	31	F	TT + CLND + LLND	PTC	1	Classic	5/37	0.14	T1aN1b	I	Intermediate
Case 3	22	F	TT + CLND + LLND	PTC	3	Hobnail	9/26	0.35	T1aN1b	I	Intermediate
Case 4	52	M	TT + CLND + LLND	PTC	9	Classic	11/32	0.34	T1aN1b	I	Intermediate
Case 5	41	F	TT + CLND + LLND	PTC	1	Follicular	14/60	0.23	T1aN1b	I	Intermediate
Case 6	21	M	TT + CLND + LLND	PTC	4	Classic with 20% tall cell	9/50	0.18	T3bN1b	I	High
Case 7	35	F	TT + CLND + LLND	PTC	2	Classic/hobnail	7/48	0.15	T1bN1b	I	Intermediate
Case 8	32	M	TT + CLND + LLND	PTC	3	Classic/follicular	14/67	0.21	T3bN1b	I	High
Case 9	28	F	TT + CLND + LLND	PTC	1	Follicular	16/28	0.57	T3bN1b	I	High
Case 10	26	F	TT + CLND + LLND	PTC	1	Tall cell	21/31	0.68	T3bN1b	I	High
Case 11	32	F	TT + CLND + LLND	**MTC**	1	-	1/26	0.04	T2N1a	I	
Case 12	51	M	TT + CLND	PTC	3	Classic	5/7	0.71	T3bN1a	I	High
Case 13	76	M	TT + CLND	PTC	1	Classic	2/5	0.4	T3aN1a	II	Intermediate
Case 14	31	M	TT + CLND	PTC	1	Classic	0/12	0	T2N0	I	Intermediate
Case 15	44	F	TT + CLND	**MTC**	1	–	3/15	0.2	T1aN1a	I	
Case 16^**a**^	59	F	TT	PTC	N/A	N/A			TXNX	I	N/A
Case 17	51	F	TT	PTC	1	Classic			T1aN0	I	Intermediate
Case 18	60	F	TT	PTC	3	Classic/follicular			T3bN0	II	High
Case 19	50	F	TT	PTC	4	Classic/follicular			T1bN0	I	Intermediate
Case 20	40	F	TT	PTC	2	Classic			T3bN0	I	High
Case 21	34	M	TT	PTC	2	Classic			T1aN0	I	low
Case 22	69	F	TT	PTC	2	Classic/follicular			T3bN0	II	High
Case 23	43	F	TT	PTC	1	Hobnail			T3bN0	I	High

*TT* Total Thyroidectomy, *CLND* Central Lymph Node Dissection, *LLND* Lateral Lymph Node Dissection, *PTC* Papillary thyroid cancer, *MTC* Medullary thyroid cancer, *N/A* Not available, *TNM* Tumour, Nodes, Metastases, *ATA* American Thyroid Association, *LN* Lymph node, *LNY* lymph node yield, *LNR* Lymph Node Ratio

^**a**^The initial operation was performed 20 years ago in a different centre; detailed pathology of the first surgery couldn’t have been reached

**Table 2:** Distribution of patients’ baseline characteristics

	Results
Sex (F/M) (*n* = 23)	15/8
Median Age (years) (range) (*n* = 23)	41 (21–76)
Histology of thyroid cancer (*n* = 23)	
Papillary thyroid cancer	21
Medullary thyroid cancer	2
Type of first surgery (*n* = 23)	
TT with CLND and LLND	11
TT with CLND	4
TT	8
Median numbers of previous surgery (range) (*n* = 23)	1 (1–3)
Type of reoperation (*n* = 23)	
LLND	4
CLND	4
SLND	1
E	14
TNM (*n* = 23)	
Stage I	3 (13%)
Stage II	20 (87%)
ATA risk (*n*^a^ = 20)	
Low	1 (5%)
Intermediate	9 (45%)
High	10 (50%)
LNR median (range) (*n* = 15)	0.23 (0–0.71)
The number of tumor foci median (range) (*n* = 22)	2 (1–9)
PTC Variants (*n*^a^ = 20)	
Classic	9 (45%)
Follicular	2 (10%)
Classic/Follicular	4 (20%)
Classic/Follicular/Solid	1 (5%)
Classic/Aggressive^b^	1 (5%)
Aggressive^b^	3 (15%)

*TT* Total thyroidectomy, *LLND* Lateral lymph node dissection, *CLND* Central lymph node dissection, *LNR* Lymph node ratio, *SLND* Selective lymph node dissection, *E* Excision

^a^The evaluation of ATA risk was limited to patients with PTC, given that the initial operation for one patient occurred in another center 20 years ago, and the specific details of the pathology data were unavailable for assessment

Consequently, the ATA risk was determined for the remaining 20 patients

^b^Aggressive variants include the hobnail and tall cell variants

The median time between the initial surgery and the subsequent surgery following PB injection was 20 months, ranging from 3 months to 240. The median number of thyroid surgeries before the staining procedure was 1, ranging from 1 to 3. Eleven patients, accounting for 47.8% of the cohort, had undergone RAI treatment before PB injection (Table 3). Among the patients, 14 (60.9%) underwent E, 4 (17.4%) underwent CLND, one (4.3%) underwent SLND, and 4 (17.4%) underwent LLND (Tables 2 and 3). The median time between PB injection and initiation of surgery (after anaesthesia) was 90 (35–210) min.

**Table 3:** Treatments and follow up of patients after first surgery

	RAI-B	NOS	NOD	LOR	Last operation	RAI-A	FU (m)	Pre TSH	Pre Tg	Pre Anti Tg	Pre CT	Post TSH	Post Tg	Post Anti Tg	Post CT
Case 1	NO	1	1	Level 3	E	YES	7	13.5	78	4.3		0.07	1.03	< 1.3	
Case 2	NO	1	1	Level 6	E	YES	14	1.4	2.77	< 1.3		0.2	< 0.2	< 1.3	
Case 3	NO	1	1	Level 4	E	YES	16	0.5	1.3	< 1.3		3.2	< 0.2	< 1.3	
Case 4	NO	1	1	Level 3	E	YES	15	24.2	2.6	< 1.3		21.6	0.4	< 1.3	
Case 5	YES	1	1	Level 3	E	YES	26	0.08	< 0.2	33.2		0.1	< 0.2	< 1.3	
Case 6	NO	2	1	Level 6	E	YES	9	0.1	5.73	< 1.3		0.1	< 0.2	< 1.3	
Case 7	YES	1	1	Level 6	E	YES	7	1.12	0.42	< 1.3		0.2	< 0.2	< 1.3	
Case 8	YES	1	1	Level 6	E	YES	19	2.74	5.94	< 1.3	–	0.07	< 0.2	< 1.3	
Case 9	YES	1	1	Level 6	E	NO	29	1.9	1.3	< 1.3		0.11	< 0.2	< 1.3	
Case 10	YES	3	2	Level 2, 6	E	N/A	2	2.42	3.13	< 1.3	–	0.3	0.3	< 1.3	
Case 11	NO	1	1	Level 6	E	NO	23	1.5	–	–	72.2	1.12	–	–	5
Case 12	YES	1	1	Level 3	LLND	N/A	2	0.09	2.07	< 1.3	–	0.01	0.22	< 1.3	
Case 13	YES	2	3	Level 6,6,7	SLND	N/A	3	9.2	167	< 1.3	–	0.1	0.9	< 1.3	
Case 14	YES	2	2	Level 3,4	LLND	YES	2	141.1	49.6	< 1.3	–	85.3	3.76	< 1.3	
Case 15	NO	1	1	Level 2	LLND	NO	6	2.1			225	1.6			3.6
Case 16*	NO	1	1	Level 6	CLND	NO	3	2.13	< 0.2	26.9	–	3.0	< 0.2	4.2	–
Case17	NO	1	1	Level 6	CLND	YES	48	0.26	< 0.2	25	–	1.6	< 0.2	< 1.3	–
Case 18	YES	2	2	Level 3,4	LLND	YES	6	0.008	< 0.2	> 1000	–	0.27	< 0.2	850	
Case 19	YES	3	1	Level 3	E	YES	22	0.28	< 0.2	4	–	0.4	< 0.2	1.8	-
Case 20	YES	2	1	Level 4	E	NO	22	0.17	< 0.2	6.2	–	0.1	< 0.2	2.6	
Case 21	NO	2	1	Level 6	E	YES	25	0.1	< 0.2	59.7	–	0.2	< 0.2	8	
Case 22	NO	1	1	Level 6	CLND	YES	8	24.3	4.38	< 1.3		25.6	< 0.2	< 1.3	
Case 23	NO	1	1	Level 7	CLND	YES	6	111.1	3.75	< 1.3		53.2	< 0.2	< 1.3	

*CLND* Central Lymph Node Dissection, *LLND* Lateral Lymph Node Dissection, *SLND* Selective Neck Node Dissection, *E* Excision, *NOS* Number of Previous Surgeries, *NOD* Number of Dyed Lesions, *LOR* Localisation of relapse, *RAI-B* Radioiodine Therapy Before the Operation with PB. *RAI-A* Radioiodine Therapy After the Operation with PB, *FU (m)* Follow-Up Time in Months, *TSH* Thyroid Stimulating Hormone, *Tg* Thyroglobulin, *Anti Tg* Anti-Thyroglobulin Antibody, *CT* Calcitonin

The median size of stained lesions was 8.8 mm (4.1–15.6). Out of the 28 stained lesions, their respective locations were as follows: 2 (7.1%) in level 2, 7 (25%) in level 3, 4 (14.3%) in level 4, 13 (46.5%) in level 6, and 2 (7.1%) in level 7 (Tables 3 and 4). The median stained lesion per patient was 1 (ranging between 1 and 3).

**Table 4:** Features of lesions stained with patent blue

Lesions	Results (*n* = 28)
Median size mm (range)	8.8 (4.1–15.6)
Location	
Level 2	2
Level 3	7
Level 4	4
Level 6	13
Level 7	2
Median stained lesion per patient (range)	1 (1–3)
Successfull resection rate	28/28

**Fig. 2:**
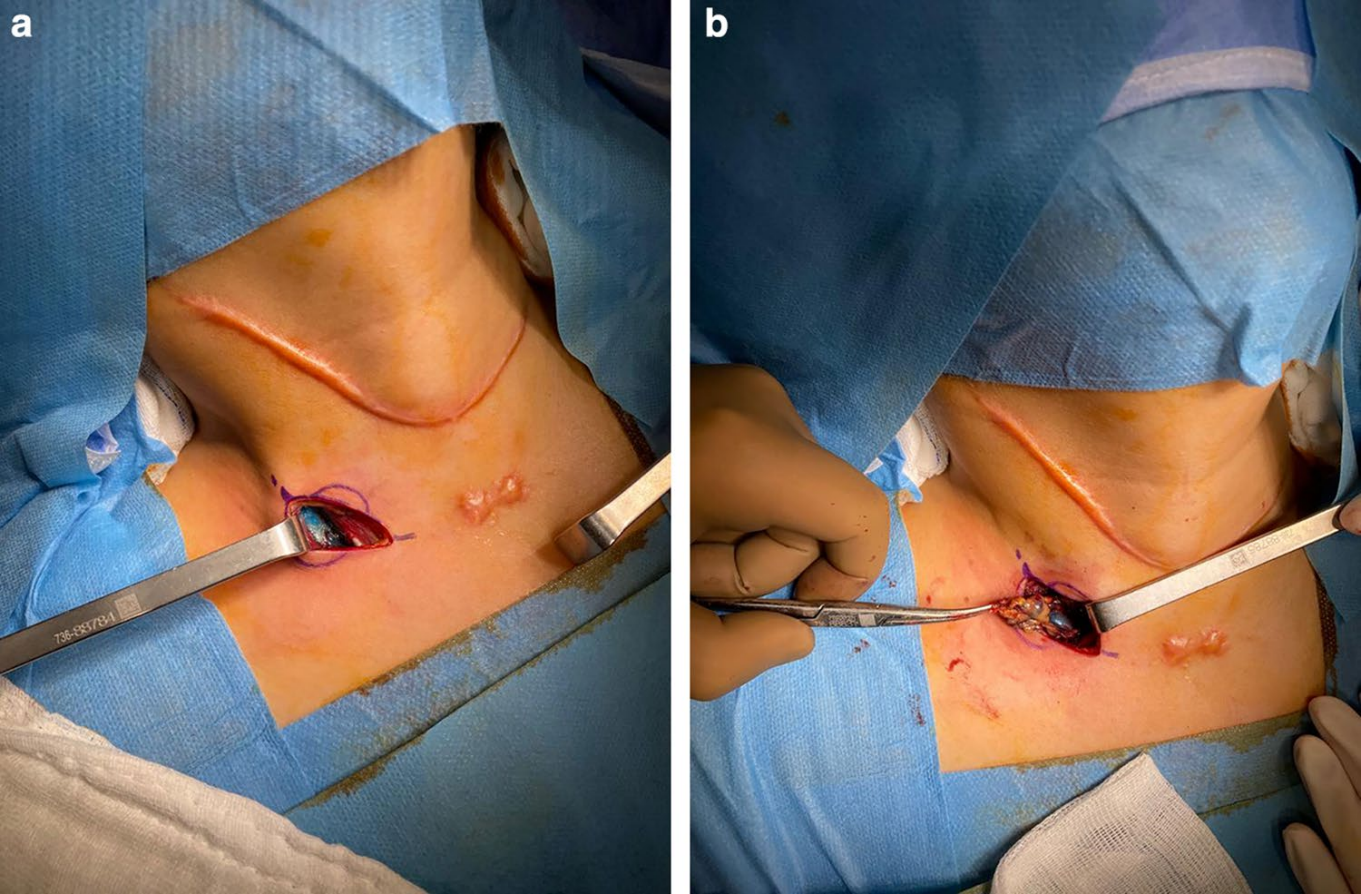
**a**, **b** Patent blue-dyed lymph node was conveniently found in the operative field

**Fig. 3:**
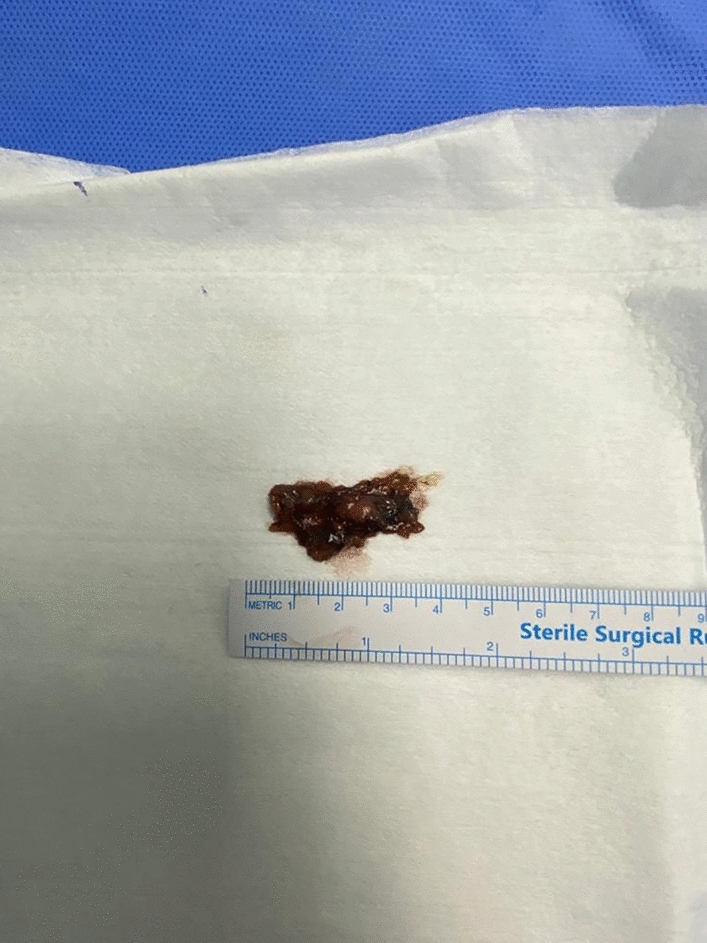
Small amount of blue dye extravasation into the surrounding tissues

During the surgical procedure, the pathological lymph node exhibited a blue colour in all instances (Fig. 2a and b). A small amount of blue dye extravasation into the surrounding tissues, which did not impede the accurate removal of the lesion, was observed (Fig. 3). The final pathology analysis verified that all resected lesions encompassed the index lesions. No complications such as anaphylaxis, intolerable pain, bleeding, nerve damage, skin discolouration, or skin/fat necrosis at the injection site were experienced by any patients. The presence of the PB did not hinder the pathologist's ability to assess or interpret the specimen.

The median duration of follow-up after the surgery with the staining procedure was nine months (range: 2–48). The lymph node that was stained preoperatively was not visualised in the US in any of the patients during the follow-up period. Although preoperative and postoperative TSH levels exhibited similarity (*p* = 0.058), a noteworthy reduction in postoperative Tg levels was observed (*p* < 0.001) (Table 3). In addition, postoperative Anti-Tg significantly decreased compared to preoperative Anti-Tg levels (*p* = 0.018). Despite the limited number of patients with MTC, a notable and significant reduction in CT values was observed. RAI was administered to 15 patients post-surgery following PB injection. RAI was not administered to 5 patients, and the status remains unknown for three patients due to a lack of extended follow-up (Table 3).

## Discussion

Our research findings indicate that the utilisation of PB dye for localising recurrent tumours, even in small lesions, in scarred reoperative neck surgeries is a technique that is both safe and efficient, yielding highly effective results. Due to the prolonged efficacy of PB dye, the lesions remain identifiable even when applied hours before the operation. Consequently, this procedure does not extend the preparation period for the surgery in the operating room.

When possible, surgery is considered the primary treatment for neck recurrences of thyroid cancer and offers the greatest likelihood of curing the patient. As surgery continues to be the most effective treatment approach for recurrences, various efforts have been undertaken to develop techniques that enhance the safety and efficacy of reoperations. Considering the importance of preserving critical tissues during the removal of recurrences within the surgical area, it is crucial to perform accurate localisation studies. These studies are necessary to differentiate between metastatic tissue, scar tissue, and the vital structures that need to be protected. Preoperative imaging, which includes US mapping [17], is crucial for precisely identifying the position of abnormal lymph nodes and strategising the approach for subsequent reoperation. Although US mapping is valuable, it does have certain limitations. Utilising marks on the skin can assist in identifying the optimal placement for making incisions. However, once subplatysmal flaps are raised and dissection is underway, the skin ceases to serve as an accurate reference point for marking the precise location of the involved lymph node. Intraoperative US may improve the identification of recurrent thyroid cancer [19]. However, relying on the US after making an incision in the neck is more challenging and less precise. It frequently extends the duration of the surgical procedure. WGL of a suspicious nonpalpable lesion before surgical removal is routinely used in the treatment of breast cancer [31]. The introduction of WGL also aimed to focus the dissection specifically on the affected lymph nodes in thyroid cancer [20, 32, 33]. Recently, Carrillo et al. compared the outcomes of WGL resection and the conventional procedure. WGL surgery for non-palpable loco-regional recurrent thyroid cancer yielded improved results in terms of surgical morbidity, recurrent/progressive disease, and recurrence-free survival [34]. WGL requires coordination between the interventional radiology and surgery services. Although this technique has advantages, it can be challenging to maintain the needle securely in place, particularly in superficial areas. In addition, positioning the needle becomes more complicated when mobilising subplatysmal flaps to expose the thyroid bed.

Additional techniques have been developed to aid in localising cervical nodal metastases, including administering radioiodine followed by surgery using an intraoperative probe (radio-guided occult lesion localisation (ROLL)) to detect suspicious lymph nodes [22, 23]. A recently published study [24], comprising the most significant patient cohort to date (*n* = 204), demonstrated a reliability of 96.57% in individuals who underwent ROLL. Challenges arise in this technique due to variations in radioiodine uptake among the tumours, leading to the potential for both false positive and false negative results. Radioactive seed localisation (RSL), initially utilised in procedures like breast cancer, lung cancer, and sarcoma resections, has now found application in thyroid and parathyroid tissues [25]. Limited case series, with the largest involving a maximum of 6 patients diagnosed with thyroid cancer, indicate successful resection of the targeted area along with the removal of the radioactive seed [26]. However, implementing RSL comes with precautions, emphasising the necessity for constant awareness of the seed's location. Surgeons use a gamma probe, and the radiology and pathology teams employ a NaI detector. After transportation to the pathology laboratory, each specimen undergoes frozen-section analysis with the NaI detector pinpointing the seed's exact location. The seed is then retrieved and processed in nuclear medicine. Handling precautions involve avoiding direct contact and refraining from intraoperative suction to prevent inadvertent seed loss. Challenges include regulatory requirements of controls by an institution's radiation safety officer. Successful procedure implementation necessitates close coordination among various specialities, including surgeons, radiologists, nuclear medicine physicians, and pathologists. Institutions intending to adopt this technique must engage in meticulous planning.

Blue dyes are used for various applications in the operating room, including sentinel lymph node localisation in breast cancer and melanoma, and to aid in identifying injuries to structures such as the ureter. These dyes are all considered safe for use in humans. There is a scarcity of studies explicitly focusing on identifying the localisation of thyroid cancer recurrence. In a study including 11 patients with thyroid cancer conducted by Ryan et al., it was shown that surgeon-performed US-guided intraoperative methylene blue (MB) injection was especially beneficial in guiding the surgeon towards the precise area for tumour resection in fibrotic regions [35]. The study conducted by Harari et al. provided evidence of the safety and effectiveness of using intraoperative US-guided injection of MB for thyroid cancer localisation and removal in 44 patients who undergo reoperative neck surgery [21]. Recently, Koca et al. demonstrated that the injection of MB, even if applied preoperatively, proved to be a safe and effective method for identifying recurrent tumours in 8 patients [36]. The average time between MB administration and the moment the surgeon could visualise the blue-stained lesion was 65 min, ranging from 57 to 71 min. Due to the utilisation of PB as a dye in our study, we could visualise the lesion even after a staining period of 210 min. Our study is the first to report the preoperative administration of US-guided PB for localising thyroid recurrences. PB exhibits a prolonged marking effect, as it has been observed to remain highly visible even after 980 min in breast lesions [37]. Injecting PB outside the operating room preoperatively, rather than during the surgery itself, reduces the duration of the stay in the operating room, where the maintenance of sterile conditions is necessary. In addition, there was no need for the endocrinologist and her equipment to be in the operating room. One initial concern associated with this method was the potential leakage of the dye, which could hinder the technique's success if the surgery was not promptly performed following the dye injection. In the current study, a patient underwent surgery 210 min after the dye injection. Remarkably, the dye remained localised at the injection site and disseminated minimally to the surrounding tissue, guiding and enabling sufficient resection. Initial studies investigating the staining and diffusion characteristics of different dyes have determined that PB is the most optimal dye for marking such lesions [37]. It exhibits sufficient diffusion, enabling safe margins without excessive dissection of neighbouring tissues.

Preoperative charcoal injection into the recurrent lesion is also an effective and safe method with results similar to our study [27, 28]. To be suitable for injection, activated charcoal requires undergoing specific processes. Compared to PB, charcoal might be less practical due to the convenience of readily available solutions offered by the latter. While it was possible to perform a superficial injection near the vessels with charcoal, injecting the lesions in the posterior aspect of the major neck vessels was not feasible. However, US-guided dye injection is possible for any lesion with US-FNA. Furthermore, charcoal was difficult to remove during pathologic tissue processing [28]. If charcoal is applied in large quantities on superficial lesions, it can result in persistent staining on the skin [27].

In our study, no adverse reactions to the PB were observed. In previous literature, allergic reactions when using PB dye have been reported to range from 0.06% to 2.7%, with an average occurrence of 0.71% [37]. The frequency of allergic reactions is primarily associated with surgery involving sentinel lymph node screening, which typically requires a larger amount of dye, usually 2 to 4 mL. Conversely, our study used a smaller volume of PB dye, specifically 0.3–0.5 mL, for marking the lesions. In addition, we did not find any case of allergic reactions in the literature during the marking of impalpable lesions with PB. A precaution that should be taken is to avoid performing the procedure in patients with a significant allergic history, such as severe urticaria and angioedema.

Despite the median lymph node size in our study being 8.8 mm, certain patients demonstrated smaller sizes, reaching as low as 4.1 mm. As recommended,

we diligently monitor individual small lymph nodes exclusively during our follow-up [7]. The staining process is applied specifically to these diminutive lymph nodes to aid the surgeon in minimizing residual tumor tissue, particularly in cases involving larger lymph nodes designated for surgery. Even in instances where surgery is being conducted for the first time in that compartment, if the lymph node is small, it is stained to ensure it is not overlooked. This staining method greatly facilitates the straightforward detection and removal of these small lesions.

Although no cost study has been conducted on the localisation methods in the recurrence of thyroid cancer, the financial implications of the procedures are elevated due to the need for multiple experts, materials-related expenses, and the relatively lower success rate. The use of radioactive material is costly, requiring a nuclear medicine unit, and is often financially unfeasible for both patients and hospitals in resource-poor settings. PB, widely accessible [38], is employed in tiny quantities. Moreover, there is no necessity for an additional specialist, as the surgeon and an experienced interventionist are sufficient for the procedure. The preoperative application of PB does not extend the operation time. These factors collectively enhance the cost-effectiveness of employing PB.

Our study has certain limitations. The overall sample size is relatively small, and there are limitations in the durability of follow-up data. Our study focused on patients with PTC and MTC, excluding patients with FTC or ATC. Notably, FTC metastasises to the lymphatic system in less than 10% of cases [39], and ATC, although rare, typically results in palpable lymph node metastases, representing a minor limitation. Sustained monitoring over an extended period is essential to assess the impact of this procedure on recurrence and survival.

In conclusion, a preoperative US-guided injection of PB is a safe, readily available and highly effective technique for localising recurrent tumours, even in small lesions within scarred reoperative neck surgeries. This technique shows promise for facilitating the operative treatment of recurrent thyroid cancer.

## Data Availability

Tables 1 and 3 contain all patient data; other tables were made by grouping these data.
